# In memoriam Jonathan C. Wright (1961–2019)

**DOI:** 10.3897/zookeys.1101.81113

**Published:** 2022-05-18

**Authors:** Katalin Szlavecz, Thomas Carefoot

**Affiliations:** 1 Johns Hopkins University, Baltimore, MD, USA John Hopkins University Baltimore United States of America; 2 University of British Columbia, Vancouver, Canada University of British Columbia Vancouver Canada

The 11^th^ International Symposium on Terrestrial Isopod Biology and this special issue of ZooKeys is dedicated to the memory of our colleague, Dr. Jonathan C. Wright, who passed away on 16 December 2019. Jonathan was a scientist, educator, musician, public servant, husband, and father.

## Life and career

**Figure 1.**
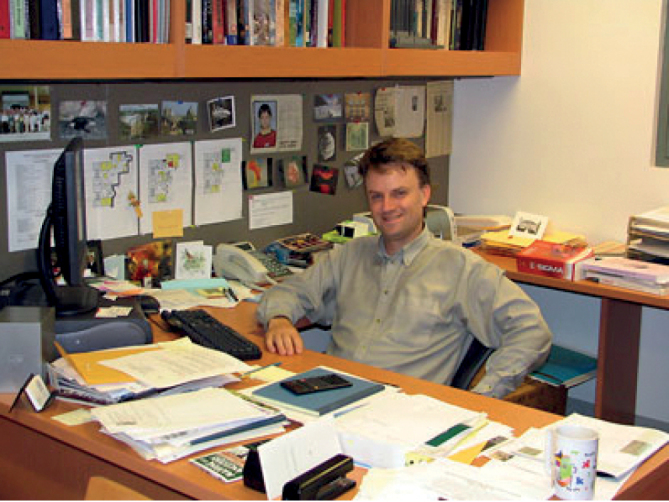
Jonathan Wright at Pomona College. Source: https://pages.pomona.edu/~jcw04747

Jonathan was born in Hull, England. His keen interest in natural history since early childhood, motivated him to study zoology, eventually earning a Bachelor’s Degree at Lady Margaret Hall in Oxford, then a Master’s and PhD at University of Oxford in invertebrate zoology. His first job was with the Nature Conservancy, then Bioscan Ltd., after which Jonathan earned several postdoctoral fellowships. He conducted research at the University of Toronto, University of Copenhagen, McMaster University in Ontario, Canada, and the Marine Biological Laboratory in Woods Hole, Massachusetts. He accepted his first faculty position at Northern State University in Aberdeen, South Dakota in 1993. Five years later, he moved to Pomona College, Claremont California, where he remained a faculty member until his death (Fig. 1). He also served as chair of the biology department in the mid-2000s, and later as Associate Dean of the College for Sponsored Research.

Jonathan’s enormous enthusiasm for science and the natural world inspired many students to conduct research projects in his lab; over the years he supervised close to 70 undergraduates, many of whom presented their findings in conferences and became co-authors in scientific publications. His passion in the subject matter, his mentorship and genuine interest in his students’ accomplishments have not gone unnoticed: Jonathan was two-time recipient of the Wig Distinguished Professor Award for Excellence in Teaching, as well as the Faculty Alumni Service Award.

**Figure 2.**
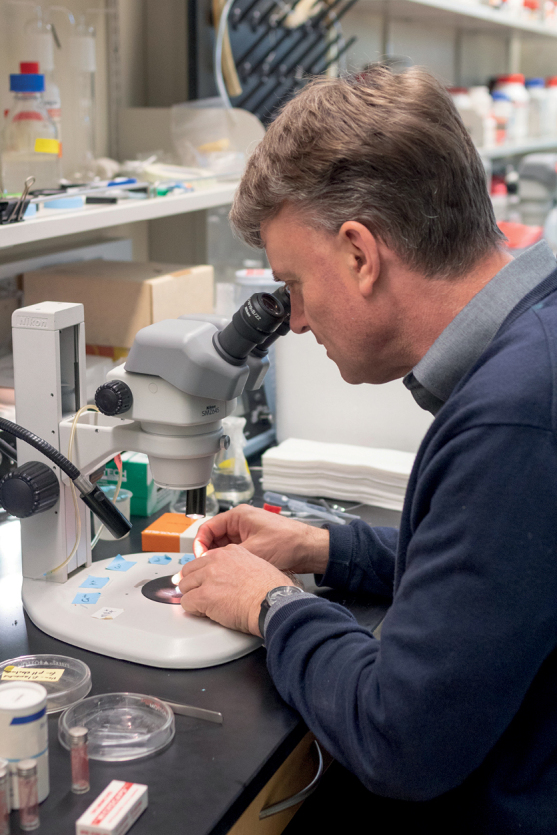
Jonathan’s research involved taking tiny amount of haemolymph from isopods. Photo: Jaysin Brandt, La Verne Magazine.

Jonathan’s research focused on ecophysiology of terrestrial isopods, specifically on water balance, nitrogen excretion, osmotic regulation and thermal balance. He conducted laboratory experiments on many species representing a range of thermal and moisture conditions (Fig. 2). These experiments required analysis of body fluids. Earlier, Jonathan and his colleagues developed a method to sample tiny (nL) amounts maxillary urine, pleon fluid, and haemolymph using a custom-made glass micropipette. Other methodological innovations included a blocking approach to locate where (mouth, rectum, pleopods) water loss/uptake occurs under different environmental conditions, and using inulin tracer to quantify the contribution of haemolymph to water loss during dehydration. These experiments required enormous focus, precision and steady hands. Recognising the importance of early developmental stages in the overall fitness of organisms, Jonathan investigated how isopod embryos in the marsupium tolerate osmotic extremes, ammonia, and pH. In the later years, he focused on patterns of nitrogen excretion, calcium accumulation, and ionic composition of juveniles while still in the marsupium.

The physiological laboratory studies were always framed in a broader, ecological/evolutionary context, and often complemented with field studies. Comparison of species from hygric to xeric habitats revealed the relative importance of water and osmoregulatory mechanisms under different thermal and moisture stress. One study on littoral isopods showed that the discontinuous ammonia excretion is constrained by the tidal cycle in *Ligiaoccidentalis*, while in *Alloniscusperconvexus* and *Tylospunctatus*, diurnal cycle regulates the same process.

As a keen zoologist, Jonathan valued ‘simple’ observations as they are often starting points of generating scientific hypotheses. Such publications or short notes do not generate much interest or citations, yet can later become significant pieces in a jigsaw puzzle. I (KS) am especially grateful for a paper, published in the Proceedings of the South Dakota Academy of Science, in which Jonathan describes winter survival of terrestrial isopods in different microhabitats in South Dakota, with its extreme winter climate. This is one of the early studies highlighting the significance of urban environments as a first step of successful colonisation of areas outside the native ranges of cosmopolitan isopods.

Jonathan’s research extended to other invertebrate groups, most notably tardigrades (Tardigrada), but he also conducted experiments on tenebrionids (Coleoptera: Tenebrionidae) and millipedes (Diplopoda). In the later years of his career, Jonathan’s focus turned to conservation efforts. His commitment to preserve natural areas and biodiversity motivated him to focus on public service and education. He served in the Claremont Hills Conservation Corporation overseeing how the city manages a nearby wilderness park. He served as a naturalist guide for Pomona College alumni in California, Alaska, and the Galapagos Island. Jonathan and colleagues were awarded two Henry David Thoreau grants to sustain local conservation efforts.

Jonathan is survived by his wife, Joanne Wright and sons Jeremy and Charlie, his mother Marjorie Wright, sister Lysetta Bray, brother David Wright, and their families. Outside teaching and research, Jonathan was an accomplished musician. He sang in the Pomona College Choir, played the violin in the Pomona College Orchestra, but also performed recitals in a string quartet. Many years ago, I (KS) visited Jonathan in Pomona. He showed me the impressive collection of isopod colonies he kept in the lab, and the experimental setup to conduct these painstaking experiments. We had lunch with Joanne and Jeremy, and then they invited me to their home. With violin and sheet music everywhere, it was evident that music was another important part of Jonathan’s life.

Jonathan regularly participated in our symposia, presented and published results on his and his students’ research. As a manuscript reviewer, he offered detailed, constructive criticism to the authors. He also kindly edited the language, greatly improving the quality of the manuscript. With Jonathan’s untimely passing, the small, close-knit community of terrestrial isopod researchers lost a great colleague.

## Reminiscences of happy times

**Figure 3.**
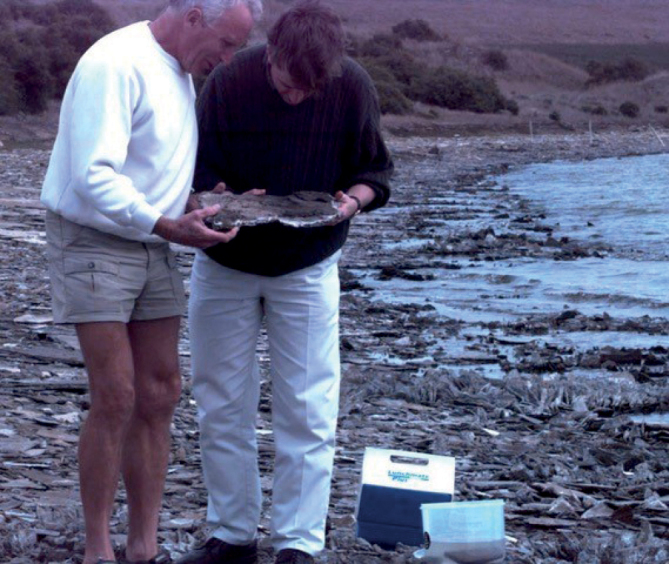
Jonathan (on the right) and Tom Carefoot looking for a *Haloniscussearlii* neat Melbourne, Australia. Photo: courtesy of THC

We thought that a story or two about my (THC) several research trips with Jonathan might be a nice addition to this obituary. One such trip was to Australia to find a saline lake near Melbourne that Jonathan had read in some Australian Naturalist’s Bulletin as a place to find the uniquely adapted isopod *Haloniscussearlei* (Fig. 3). Despite my misgivings and with credit to Jonathan’s sure-minded enthusiasm, we were successful, and also later collected *Ligiaaustraliensis* to study their desiccation resistance.

That trip was followed the next year by several days at the Bamfield Marine Sciences Centre on the west coast of Vancouver Island, where we spent a night on a wild beach in a tiny tent. Jonathan stayed ensconced in relative comfort in the tent while I went out every two hours over a full day and night to collect *Ligiapallasii* for haemolymph sampling. In preparation for the stay we ensured that we had all we needed for every contingency. But when day turned to night as we prepared for much-needed sleep, I said to Jonathan: “one problemo, no alarm clock”. He replied that it was no problem, he had a built-in alarm.... Sure enough, every two hours through the night on the dot I felt him nudge me and off I went, thinking to myself who is this superpowered man?

Two years later we were in Hawai’i with two species on our minds, *Ligiaexotica* which I knew could be collected in the Honolulu harbour area, and *Ligiaperkinsii* that had previously been found off the Pali Escarpment road. We went that evening armed with powerful flashlights to find this highly elusive species. There is not much of a story here except that it was unsuccessful. How does one find an animal that likes to inhabit moist moss on trees? Well, you find them by staring ceaselessly at a portion of moss on a tree, until you eventually see a pair of eyes looking out...then, at the smallest disturbance, they leap out and, if you are lucky, can be caught in a net. We had more success the next day in finding extra-large *Ligiaexotica* on the harbour’s surrounding high concrete walls . Jonathan took charge, with him standing at the top and reaching down 2–3m or so with a stick to dislodge them so that they fell into the water, at which time I would fetch them in a leaky old rowboat that we had scavenged. It worked like a dream until we heard a gruff, authoritarian voice yelling, “hey, you fellows, what are you doing here, you’re not allowed in the harbour, and unauthorised use of harbour vessels is not permitted!” Without missing a beat Jonathan took charge, saying something like: “it’s all right sir, we’re from the University following up on a report of an infestation of Japanese *funemushi* in Honolulu Harbour” (I taught him that word courtesy of my university Japanese 101 class). The man was old enough at least to know about the Japanese bombing, and was sympathetic, and all that Jonathan said was true: we *were* from the university and we *were* following up on a report. The fact that the report was from me made it no less true. The next day we found ourselves in a marshy area just south of Pearl Harbor, armed with Jonathan’s haemolymph-sampling equipment: a pin, capillary tubes, a clay-like plugging substance, and a piece of dry ice. We had little else: no food, no water, no flashlight, no mosquito spray, no tent, and nothing to sleep on. We had not actually planned on staying overnight, just following up advice that we should find *Ligiahawaiensis* there. Well, they were there, in abundance. So, what to do? Jonathan, our man of action, asked for the car keys and disappeared. Within moments it seemed, he was back with an armload of takeout food, flashlights, a collecting bucket, and small net, and believe it or not, a 5 × 8 foot-thick ‘foamy’ that he found at the nearby garbage dump to sleep on. Superman yes, but also Wonderman, all in one. No alarm clock, but then we didn’t need one, did we?! No mosquito spray either, but it turned out there was none in this marshy, wet, tropical swamp...for all I know arranged by Jonathan! It all went well and, at the end, we had a heart-warming mano-a-mano hug in triumph. Only later did we learn that we should have been very careful, as the area was rife with water-borne parasites. No problem!

From these and other experiences, I found Jonathan to be one of the most knowledgeable biologists I have ever had the pleasure to know, an astute “take-charge” kind of guy, and one of the best research colleagues that I have ever met. In the years following we corresponded regularly and even cooperated on a last publication for me. I miss him greatly.

## Selected publications by Jonathan Wright

Wright JC, Machin J (1990) Water vapour absorption in terrestrial isopods. Journal of Experimental Biology 154: 3–30. https://doi.org/10.1242/jeb.154.1.13

Wright JC, O’Donnell MJ (1992) Osmolality and electrolyte composition of pleon fluid in *Porcellioscaber* (Crustacea, Isopoda, Oniscidea): Implications for water vapour absorption. Journal of Experimental Biology 164: 189–203. https://doi.org/10.1242/jeb.164.1.189

Wright JC, Machin J (1993) Atmospheric water absorption and the water budget of terrestrial isopods (Crustacea, Isopoda, Oniscidea). The Biological Bulletin 184(2): 243–253. https://doi.org/10.2307/1542232

Wright JC, O’Donnell MJ (1993) Total ammonia concentration and pH of haemolymph, pleon fluid and maxillary urine in *Porcellioscaber* Latreille (Isopoda, Oniscidea): relationships to ambient humidity and water vapour uptake. Journal of Experimental Biology 176(1): 233–246. https://doi.org/10.1242/jeb.176.1.233

Wright JC, Donnell M, Reichert J (1994) Effects of ammonia loading on *Porcellioscaber*: glutamine and glutamate synthesis, ammonia excretion and toxicity. Journal of Experimental Biology 188(1): 143–157. https://doi.org/10.1242/jeb.188.1.143

Wright JC, Holliday CW, O’Donnell MJ (1994) Na+/K+-ATPase Activity in the pleopods and hindgut-rectum of terrestrial isopods: Implications for colligative water vapor absorption. Journal of Crustacean Biology 14(3): 522–529. https://doi.org/10.2307/1548998

Wright JC, Caveney S, O’Donnell MJ, Reichert J (1996) Increases in tissue amino acid levels in response to ammonia stress in the terrestrial isopod *Porcellioscaber* Latr. Journal of Experimental Zoology 274(5): 265–274. https://doi.org/10.1002/(SICI)1097-010X(19960401)274:5%3C265::AID-JEZ1%3E3.0.CO;2-N

Wright JC (1997) Winter survival and overwintering behavior in South Dakota Oniscidea (Crustacea, Isopoda). Proceedings of the South Dakota Academy of Science 76: 45–56.

Wright JC, O’Donnell MJ, Sazgar S (1997) Haemolymph osmoregulation and the fate of sodium and chloride during dehydration in terrestrial isopods. Journal of Insect Physiology 43(9): 795–807. https://doi.org/10.1016/S0022-1910(97)00035-8

Carefoot TH, Wright JC, Pennings SC, Ziegler A, Zimmer M, Uglow RF, Danford AR, Danko JP (2000) Hemolymph homeostasis in relation to diel feeding activity and microclimate in the prototypal land isopod *Ligiapallasii*. Canadian Journal of Zoology 78(4): 588–595. https://doi.org/10.1139/z99-234

Surbida KL, Wright JC (2001) Embryo tolerance and maternal control of the marsupial environment in *Armadillidiumvulgare* (Isopoda: Oniscidea). Physiological and Biochemical Zoology 74(6): 894–906. https://doi.org/10.1086/324474

Wright JC, Carefoot TH, Albers MA (2003) Osmoregulation and salt excretion in the Ligiidae and Tylidae (Isopoda, Oniscidea). Crustaceana Monographs 2: 311–334. https://doi.org/10.1163/9789047412854_023

Wright JC, Peña-Peralta M (2005) Diel variation in ammonia excretion, glutamine levels, and hydration status in two species of terrestrial isopods. Journal of Comparative Physiology B 175(1): 67–75. https://doi.org/10.1007/s00360-004-0463-z

Ouyang D, Wright JC (2005) Calcium accumulation in eggs and mancas of *Armadillidiumvulgare* (Isopoda: Oniscidea). Journal of Crustacean Biology 25(3): 420–426. https://doi.org/10.1651/C-2564

Wright JC, Ting K (2006) Respiratory physiology of the Oniscidea: Aerobic capacity and the significance of pleopodal lungs. Comparative Biochemistry and Physiology Part A: Molecular & Integrative Physiology 145(2): 235–244. https://doi.org/10.1016/j.cbpa.2006.06.020

Wright JC, O’Donnell MJ (2010) *In vivo* ion fluxes across the eggs of *Armadillidiumvulgare* (Oniscidea: Isopoda): the role of the dorsal organ. Physiological and Biochemical Zoology 83(4): 587–596. https://www.jstor.org/stable/pdf/10.1086/651583.pdf?casa_token=u_jcqtLqKGwAAAAA:8egY0CAjCoESz-ARySllAxQPI2V8Jsj3r0-3Iti7A3O0JzdhcwrFves1mJaXpU26WJtgU4-DohEREYY7Mar9pO6aL-IN3GmpbKciWmTugL1_rvrTLiw

Koh H, Wright JC (2011) Cation regulation by the terrestrial isopod *Armadillidiumvulgare* (Crustacea: Isopoda: Oniscidea) during dehydration in air. Comparative Biochemistry and Physiology Part A: Molecular & Integrative Physiology 159(2): 134–140. https://doi.org/10.1016/j.cbpa.2011.02.009

Nakamura M, Wright JC (2013) Discontinuous ammonia excretion and glutamine storage in littoral Oniscidea (Crustacea: Isopoda): testing tidal and circadian models. Journal of Comparative Physiology B 183(1): 51–59. https://doi.org/10.1007/s00360-012-0694-3

Linton SM, Wright JC, Howe CG (2017) Nitrogenous waste metabolism within terrestrial Crustacea, with special reference to purine deposits and their metabolism. In: Weihrauch D, O’Donnell M (Eds) Acid-Base Balance and Nitrogen Excretion in Invertebrates. Springer International Publishing Switzerland, 25–59. https://doi.org/10.1007/978-3-319-39617-0_2

Nako JD, Lee NS, Wright JC (2018) Water vapor absorption allows for volume expansion during molting in *Armadillidiumvulgare* and *Porcelliodilatatus* (Crustacea, Isopoda, Oniscidea). ZooKeys (801): e459. https://doi.org/10.3897/zookeys.801.23344

Rockhill E, Schammel K, Reyes-Guzman A, Harris Z, Wright JC (2019) Nitrogen excretion during marsupial development in the terrestrial isopod *Armadillidiumvulgare*. Comparative Biochemistry and Physiology Part A: Molecular & Integrative Physiology 227: 92–99. https://doi.org/10.1016/j.cbpa.2018.09.029

Wright JC, Harris ZC (2020) Aerial and aquatic respiration in littoral Oniscidea (Isopoda) from Southern California, USA. The Journal of Crustacean Biology 40(4): 462–470. https://doi.org/10.1093/jcbiol/ruaa023

Harris ZC, Wright JC (2021) The physiology of *Venezilloarizonicus* (Isopoda, Armadillidae): metabolism and thermal tolerance. Crustaceana 94(2): 159–175. https://doi.org/10.1163/15685403-bja10026

